# Enacting support for digital competence in real-life interactions when older persons receive one-to-one coaching

**DOI:** 10.3389/fpubh.2026.1771579

**Published:** 2026-02-25

**Authors:** Elisabeth Berglund Kristiansson, Pernilla Bjerkeli, Elisabeth Dahlborg, Mia Berglund, Sophie Mårtensson

**Affiliations:** 1 School of Health Sciences, University of Skövde, Skövde, Sweden; 2 Department of Health Sciences, University West, Trollhättan, Sweden

**Keywords:** digital competence, digital divide, digital support, learning environments, older persons, thematic analysis

## Abstract

**Introduction:**

Digital competence is essential in today’s digital society. Older persons often exhibit lower levels of digital competence than younger age groups, indicating a digital divide. Consequently, support to enhance digital competence is needed. Yet, little is known about the characteristics of such support. This study, conducted in Sweden, explored how one-to-one digital support was provided to older persons in their homes through a coaching service provided by a municipality.

**Methods:**

This study employed an exploratory qualitative design. Data were collected through audio-recorded support sessions in which 12 persons aged 65 years and older received support with their digital needs in their homes from a municipally employed digital coach. The audio-recorded sessions were analysed using thematic analysis.

**Results:**

Through the analysis, three main themes emerged, each comprising two sub-themes. The themes were direct support and memory aid, provided through hands-on support and tools for memory support; support built on relation and mutual influence, achieved by building a relationship and creating conditions for equal voices; and to place the person’s own digital needs at the centre, accomplished by listening and responding and by sharing adapted knowledge. The support was provided through the creation of an adapted and inclusive learning environment centred on the older persons’ own digital needs.

**Conclusion:**

The study provided insights into how support was enacted in real-life interactions when older persons received coaching for digital competence in their own homes. These insights may contribute to improving the design of future interventions. They also highlight the potential for digital support to narrow the digital divide and promote equitable access to the resources of the digital society.

## 1 Introduction

Digital competence is essential in today’s digital society, and its absence contributes to the digital divide, increasing the risks of social exclusion and health inequalities (1–3). Older persons, like other citizens, are required to engage with digital technologies as part of everyday life, including maintaining social relationships, managing financial affairs, interacting with public authorities, and ensuring digital security (4). However, older persons often have lower levels of digital competence compared to younger age groups (4, 5), and are therefore at increased risk of exclusion and loneliness, which may in turn have negative health consequences (5). The need for support to increase digital competence among older persons is, therefore, substantial. Societal initiatives are being increasingly directed towards addressing this issue, reflecting a growing recognition of its significance (6). However, knowledge about how this support is best designed to promote the development of digital competence in older persons remains limited (7). Therefore, there is a need to deepen the understanding of the design and content of such support in order to effectively strengthen digital competence among older persons.

The concept of digital competence is formally defined by the Council of the European Union as a key competence for lifelong learning (8). Digital competence refers to the confident, critical, and responsible use of digital technologies for learning, work, and societal participation. This includes the ability to manage digital information, communicate securely and ethically, and use digital tools to support collaboration, inclusion, and well-being. Individuals are expected to understand the principles and functions of digital technologies, including their opportunities, limitations, and societal and health-related impacts, and to apply them reflectively to promote safe, informed, and inclusive digital engagement (8). A comprehensive framework, DigComp, has been developed to support the practical implementation of this definition, providing guidance for its application across different contexts such as policy, research and education (9–11). Although the framework was not applied in this study, which focused on analysing the service as provided in practice, it is relevant for understanding efforts to strengthen citizens´ digital competence, including digital confidence, digital skills and exploiting the benefits of digitalisation.

Compared to younger age groups, older persons generally exhibit lower levels of digital competence, partly due to infrequent internet use and limited exposure to digital technologies, as well as a lack of prior experience and perceived relevance of digital devices in daily life (12–14). In the European Union (EU), only 25% of women aged 65–74 years have basic digital competence, compared to 71% of those aged 25–34 years. Among men, the figures are 34% versus 69% (15). Internet use tends to decrease progressively with advancing age and, consequently, so does digital competence (16). This highlights a generational divide in digital competence and a public health concern, as it may impede older persons’ access to health information and health services, thus posing challenges for health equity and digital inclusion (14, 15). However, variations in digital competence are evident even within the older population. Individuals with higher levels of education, greater economic resources, and stronger cognitive abilities are more likely to engage with digital technologies. These factors contribute to disparities in digital inclusion among older persons (17). Moreover, barriers to digital competence among older persons include limited technical knowledge, difficulty understanding digital terminology, physical limitations, uncertainty about how to use digital technology, and a perceived lack of support. Additionally, many older persons find digital devices irrelevant to their everyday lives, which affects their motivation to develop their digital competence (18, 19).

To address the challenges faced by older persons in improving digital competence, specific forms of support become essential (7). One such form is coaching, defined in accordance with the principles of Fletcher and Mullen (20) as a structured, one-to-one supportive relationship between a coach and an older person, with the purpose of enhancing the older persons’ digital competence. Providing support to older persons with their issues related to digital technology involves addressing the person, their specific digital needs, and the technological problems themselves (7, 18, 21). The interaction with the older person, during which support for their digital needs is provided, is influenced by the communication between the parties (7, 18, 22). Communication consists of various components, often involving interaction during the meeting and the conversation (23), which are important to consider for the success of the support (24).

Providing appropriate support to older persons, who may have varying levels of digital competence, requires an understanding of how to offer support that ensures equitable access to the resources of the digital society (7, 25, 26). An important aspect of adequate support for older persons’ needs is understanding the level or levels of digital competence at which the individual describes their problems and desires.

The level of digital competence reflects a digital divide in society, generally understood across three levels: the first concerns access to the Internet and digital devices, the second involves the ability to use them, and the third relates to benefiting from the opportunities they provide (2). Considering the heterogeneity of older persons’ needs and backgrounds is, therefore, essential for providing adequate support. Failing to do so risks contributing to digital ageism, an emerging form of discrimination at both individual and societal levels, which reduces the possibility of equal access to the digital society (3, 27, 28).

Support aimed at improving older persons’ digital competence must be adapted to ensure it is accessible and provides meaningful outcomes (21). From a socio-cultural perspective, older persons’ learning can be understood as part of lifelong learning and often reflects their current needs and desires, as well as the knowledge, abilities, and limitations that a long life may bring (22, 29). Learning can take place in a variety of settings, such as public venues, educational institutions, or in older persons’ own homes. Previous research has suggested that older persons value the opportunity to learn at home (7, 30, 31). The home is a familiar environment where their own digital technology is available (7, 30, 32) and a place where individuals are expected to be given the opportunity to age in place (33).

One-to-one support is the preferred form of support among older persons for various reasons, including uncertainty in using digital technology, differing levels of digital competence, and limited energy for acquiring new knowledge (7, 21). In Sweden, several municipalities have, therefore, begun providing different forms of home-based one-to-one support. Despite the demand for such support and the efforts undertaken, there remains a lack of comprehensive knowledge in the literature regarding how support should be designed to enable older persons to develop and enhance their digital competence. Consequently, no best practice has yet been established (7).

To adequately address older persons’ expressed need for enhanced digital competence, it is imperative to investigate the modalities and content of the individualised support provided in their homes. Therefore, the aim of this study was to explore and describe how one-to-one digital support was provided to older persons in their homes through a coaching service provided by a municipality.

## 2 Materials and methods

### 2.1 Study design

A qualitative design with a focused ethnographic approach was employed (34). Audio recordings of sessions in which older persons received in-home support for their digital needs from a digital coach were analysed using reflexive thematic analysis (35, 36).

### 2.2 Setting and participants

The study was conducted in a medium-sized Swedish municipality. The Digital Coach Service was funded and operated by the municipality and existed independently of the present study. At the time of data collection, a few municipalities had implemented similar initiatives. The service is advertised via the municipality’s website and in the local newspaper and is available to all residents aged 65 and older. It is free of charge and can be used multiple times. Older persons contact the municipality themselves to request a visit from a digital coach.

Initially, contact was made with the municipality’s Public Health Sciences Unit, which coordinated communication among the digital coach, the municipality’s information technology department, and the researchers (EBK and MB). Convenience sampling was applied (37), determined by occasions when both the researcher (EBK) and the digital coach were available to conduct in-home support visits. Persons aged 65 years and older who contacted the municipality for support from a digital coach and were able to speak and understand Swedish were included.

Recruitment began when an older person contacted the digital coach to request support. Following the initial request to participate, the researcher (EBK) contacted each participant and provided information about the study, both verbally and in writing.

In total, 14 persons were invited to participate. Of these, 12 (five women and seven men), aged between 68 and 92 years, consented to take part. The participants’ homes were located either in the municipality’s city or a few kilometres outside it. Most lived alone, with only three residing with a spouse.

The support sessions were conducted in the older person’s home in a room where their digital devices were placed on a table. During the sessions, the older person and the digital coach sat next to each other, while the researcher (EBK) was seated a few metres away.

### 2.3 Data collection

Data collection took place during June–December 2021. Each support session lasted approximately 1.5 to 2 h and was audio-visually recorded using a camera operated by the researcher (EBK). For this study, only the audio component was used, specifically the voices of the older persons and the digital coach, which were transcribed and analysed. The researcher (EBK) adopted a non-participatory approach during the sessions, positioning themselves to observe and record the ongoing support activity while hearing the conversation between the older person and the digital coach. Observing on site allowed the researcher to witness the coaching session directly, rather than relying solely on the recorded audio.

### 2.4 Data analysis

The analysis included audio recordings of 12 support sessions conducted by the same digital coach with 12 different older persons. The recordings were transcribed verbatim and aggregated for analysis using reflexive thematic analysis, as outlined by Braun and Clarke (35, 36). Reflexive thematic analysis seeks to describe a subject in a condensed manner by identifying how different codes relate and cluster together. This flexible method was chosen for its ability to systematically identify and analyse patterns (themes) within qualitative data. The analysis process followed the method’s six phases, with a deliberate focus on the researcher’s reflexivity to account for the subjective nature of preunderstanding and interpretation.

The researcher’s (EBK) presence during data collection contributed to contextual understanding, which was acknowledged as part of the reflexive thematic analysis process (36) and the focused ethnographic approach (34). The analysis began with familiarisation with the data, which started during data collection and continued when audio files were extracted from the audio-visual recordings prior to verbatim transcription. The researcher (EBK) repeatedly listened to the audio files and read the transcripts, recording reflections as comments in the text documents. This initial analysis was discussed with the co-authors to ensure a shared understanding of the data. Once a comprehensive sense of the material was established, the data were examined to derive codes related to the study’s aim, which were compiled in an overview table. These codes formed the building blocks of the analysis. To enhance trustworthiness (35, 36), the researcher (EBK) reviewed the codes through analytical and reflexive discussions with the co-authors.

Through active and engaged reflection by EBK, in collaboration with the co-authors, initial themes were generated from the coded data. These themes were subsequently discussed and further refined together with the co-authors. EBK reviewed the initial themes in relation to the text and codes, further developing them through an increasingly interpretive analysis and ongoing discussion with the co-authors.

In the penultimate phase, an active and iterative process was undertaken to refine the themes in order to better illuminate and convey the overarching narrative. This involved ongoing discussions with the co-authors to carefully consider and adjust the theme names, ensuring they accurately reflected the evolving understanding of the data. Sub-themes were also assigned descriptive names.

Finally, during the writing of the results, a last opportunity was taken to review the analysis, themes, sub-themes, text, and quotations to ensure their relevance to the study’s aim. Consistent with reflexive thematic analysis as described by Braun and Clarke (35, 36), the analytical process was not linear but iterative, involving continuous movement between the whole text and its parts.

### 2.5 Ethical consideration

This study was approved by the Swedish Ethical Review Authority (Dnr 2021-03524) and conducted in accordance with the World Medical Association Declaration of Helsinki (38). The participants received both oral and written information about the study, including that participation was voluntary and that they could withdraw at any time without providing a reason or facing any consequences. Written informed consent was obtained from all participants.

All collected data have been coded, anonymised, and securely stored. These include the audio-visual recordings and physical materials, as well as audio files and transcribed texts, in designated data storage locations.

## 3 Result

The study results explored how digital support was provided to older persons in their homes by a municipally employed digital coach. Through thematic analysis (35, 36), three main themes emerged, each comprising two sub-themes illustrating different characteristics of the digital support. The themes were as follows: direct support and memory aid, support built on relation and mutual influence, and to place the person’s own digital needs at the centre. The themes and sub-themes are presented in Table 1. Each theme and sub-theme are described in detail below, accompanied by quotes from either the older person (OP), the digital coach (DC), or both.

**Table 1:** Themes and sub-themes describing how digital support was provided to older persons in their homes by a municipally employed digital coach.

Themes	Sub-themes
Direct support and memory aid	Hands-on support Tools for memory support
Support built on relation and mutual influence	Building a relationship Creating conditions for equal voices
To place the person’s own digital needs at the centre	Listening and responding Sharing adapted knowledge

### 3.1 Direct support and memory aid

The first theme addresses the basic prerequisites for developing digital competence and includes two sub-themes: ‘hands-on support’ and ‘tools for memory support’. This theme relates to restoring the functionality of digital devices to ensure that they can be accessed and used effectively. Support involved hands-on support with troubleshooting digital hardware and software on the older persons’ own devices and applications. It also included the creation of tools for memory support, aimed to facilitate memory retention and enable individuals to independently replicate digital tasks in the future.

#### 3.1.1 Hands-on support

Hands-on support included practical assistance with digital issues that the older person had identified as needing help. The most common hardware requiring support included computers, printers, mobile phones, and televisions, along with the associated software. Frequently requested applications included music and film applications, social media, email, SMS, Swish (a mobile money transfer application), and BankID (a digital identification system). Support was also provided for tasks such as installing a virtual private network (VPN) or free office software.

The support sometimes involved simply ‘fixing’ a digital problem, meaning that the digital coach resolved an issue that usually required attention only once. During this process, the digital coach often engaged the older person by continuously explaining what was being done. The following quote illustrates a situation in which a repair tool needed to be downloaded to the older person’s computer, and the digital coach addressed the problem.

*“DC: …control panel, because we were inside here on the … page … The NET Framework… It recommends that we download Microsoft repair… OP: Yes, a repair tool… It's just that I can't get it in… DC: Exactly … we can check …”.* (Participant 8)

The older person could thus follow what was happening, even though it was the digital coach who ‘fixed’ the digital problem. This provided an opportunity for the older person to learn from the support. Hands-on support also included instances in which the digital coach and the older person collaboratively addressed a digital problem, with the practical implementation carried out by the digital coach.

*“OP: … Downloading apps … it’s been a bit … I tried, but I just stopped… it never really got going… so to speak, I haven’t been able to create any [app] and that is really what is … getting help with Swish … DC: Exactly, if we test then … OP: … install it in that case…DC: Exactly…”.* (Participant 7)

This demonstrates that hands-on support from the digital coach was necessary to install the desired application, enabling the older person to use it. Another aspect of hands-on support involved providing explanations and answering the older persons’ questions related to their digital needs.

*“OP: But I will also ask, when you get the prompt “press Enter” … DC: Do you get it at …? OP: Yes, it says here then … press Enter, then …what is Enter …? DC: Enter on the keyboard is the big square… OP: The one you have for spaces…? DC: There, not the space bar, but there… will see if I find it on (writes) Enter key… there… OP: So that's Enter!”* (Participant 5)

Through hands-on support, the person was given an opportunity to develop an understanding of the keyboard layout.

#### 3.1.2 Tools for memory support

Tools for memory support included various strategies aimed to help the older person remember what was discussed and demonstrated during the support session, enabling them to perform the tasks independently later. For example, older persons often requested that memory notes be written during the session, and the digital coach also recommended using a memory book for storing passwords.

The memory notes were written to help the older person remember instructions for digital tasks and to provide an opportunity to repeat the activity at a later time. One example involved instructions on transferring a photo from a mobile phone to a computer.

*“DC: … and then it's that you connect… should I write? OP: Yes, please. You know, afterwards when you've left, then what on earth was it I actually answered to? DC: Exactly… (DC writes) … pictures … phone to computer … it should be … plug in… mobile … into the computer … with connection cable … and then it is … mobile …”.* (Participant 1)

A password memory book was created to help the older person remember the various passwords required for multiple digital logins. This emerged as a memory-supporting tool, designed to ensure the retrieval of authentication details in the future.

*“OP: And then we'll write down what it means, so I have it in writing… DC: …exactly, … I usually recommend that you keep a small book at home with passwords …”.* (Participant 11)

The written memory aids, reinforced by the digital coach, were important for remembering passwords and served as tools to support memory.

### 3.2 Support built on relation and mutual influence

The second theme included two sub-themes: ‘building a relationship’ and ‘creating conditions for equal voices’, reflecting aspects of support shaped through personalised interaction in which both parties respectfully allowed each other space to speak. This theme highlights that building a relationship during the support session was a prerequisite for the older person to reveal their digital needs and for the digital coach to understand the needs expressed. Small talk during short breaks facilitated interpersonal connection and mutual understanding of digital needs. Additionally, when the digital coach acknowledged and praised the older person’s existing digital competence, it created an opportunity to strengthen the person’s sense of being knowledgeable. The interaction between the older person and the digital coach were characterised by alternating control over the support agenda, fostering conditions for equal voices and enabling mutual influence and shared decision-making.

#### 3.2.1 Building a relationship

Building a relationship appeared to be important for the older person to feel confident in receiving support. The digital coach and the older person met for the first time, during which the older person was exposed by revealing their limited digital competence to a stranger. Small talk was common and contributed to a relaxed atmosphere. Furthermore, recurring acknowledgement and praise provided the older person with reassurance that they were on the right track in developing their digital competence.

The small talk covered a range of topics, with older persons discussing their hobbies, such as painting, participating in sports, travelling, or doing volunteer work. It was often related to the immediate situation. For example, one older person described, during small talk, the challenges of getting to the local bank.

*“OP: Because the bank is so poorly positioned, it has moved down from the crossing and it's so stupid… DC: Yes, over there by the university… OP: Yes, and then you must park in the large parking garage, it's so complicated, I think… I've got a new security token… DC: You can log in with the mobile BankID as well”.* (Participant 6)

This small talk addressed local parking issues but was also related to the need to use a digital identification system. It reflected a dynamic approach to presenting an issue within a context that appeared meaningful to the older person.

A short and confirmatory word frequently used by the digital coach was ‘exactly’. This term was employed when the older person referred to issues or observations related to digital matters. By using this word, the digital coach provided a brief but important signal, confirming to the older person that their reasoning was on the right track. Furthermore, the older persons were praised for their own solutions and achievements.

*“DC: … so then it's good you've already fixed … things like the screensavers and that kind of stuff … that it goes out and … it works as it should… Yes, that's good”.* (Participant 3)

The statement conveyed positive feedback on the older persons’ digital knowledge and abilities. Praise was frequently offered, often directly linked to the issues discussed during the support session.

#### 3.2.2 Creating conditions for equal voices

During the support session, conditions for equal voice were created by both the older person and the digital coach. This provided opportunities for mutual influence on the support, as well as space for new questions, challenges, and ideas to be discussed, contributing to a permissive atmosphere. The following situation exemplifies how an older person shifted to another topic during the ongoing interaction.

*“DC: I'm thinking if you get a message about this with Inc [the ink cartridge store], let me know… OP: Okay, … then I ask, I don't know if you have time, but I can't get the emails on my tablet… DC: Exactly … it is possible …”.* (Participant 6)

The digital coach respected the older person’s wish to redirect the support towards a new, specific digital need. Similarly, the digital coach sometimes changed the subject during ongoing conversations, thereby steering the support in a new direction. By creating conditions that ensured equal voice, opportunities for mutual influence were established, which, in turn, enabled the support to develop throughout the session.

### 3.3 To place the person’s own digital needs at the centre

The third theme included two sub-themes: ‘listening and responding’ and ‘sharing adapted knowledge’. The support was provided with the older persons’ digital needs at the centre and was expressed by both parties through respectful listening and by responding to questions and reflections. The support was also facilitated by the digital coach continuously sharing knowledge adapted to the older persons’ digital needs during the ongoing activities. The older persons demonstrated a willingness to reveal gaps in their knowledge, to actively pose questions, and to use digital terminology such as ‘edit contact’ and ‘internet browser’. Such openness indicates a readiness to engage in learning processes. This further suggests that both listening and responding and sharing adapted knowledge were valuable characteristics in addressing digital challenges and fostering digital competence.

#### 3.3.1 Listening and responding

The digital coach and the older person listened carefully to each other during the support session and responded attentively. This was achieved by verbally articulating topics of relevance, paraphrasing what was said, and providing timely feedback. This approach appeared to enable answers to be aligned more closely with the needs verbally expressed by the older person. The following quote illustrates how the digital coach listened and paraphrased a question about a digital issue posed by the older person, while simultaneously drawing attention to the subsequent clarification provided.

*“OP: What does that edit contact mean? DC: Exactly, that's if you're inside contacts… OP: Yes but … edit? DC: …that's … you have Bosse and his phone number so you can edit and write Bosse and his last name… OP: Well, then I know what it is!”* (Participant 5)

This interaction illustrates how the digital coach’s attentive listening and paraphrasing prompted the older person to realise that the initial question had been only partially addressed, leading to further clarification. In turn, this careful listening enabled the digital coach to provide a response that more precisely addressed the clarified question. In the next quote, the digital coach verbally describes the handling of the older person’s computer, ensuring that the support aligned with the person’s needs and preferences. The older person’s willingness to listen and respond to what the digital coach verbally shared and the digital coach’s attentiveness to the older person’s responses emerged as important for making shared decisions about further action.

*“DC: Then we'll see, then we have autostart here, then we have Skype here, booted up when the computer starts up and it doesn't have to do that… But now we'll see, then you have something else here, Cortana for example, does not have to boot when the computer starts either… OP: What kind of thing is that? DC: It's a voice assistant, though it doesn't work on computers outside the U.S. OP: Oh, my goodness, take it off!”* (Participant 12)

This interaction exemplifies how the digital coach’s verbalisation of actions facilitates a collaborative decision-making process. By actively listening and responding to the older persons’ input, the digital coach enables them to retain control over their digital devices. In this way, the practice of listening and responding supports the process by placing the older persons’ digital needs at the centre.

#### 3.3.2 Sharing adapted knowledge

Sharing adapted knowledge also helped to place the older person’s own digital needs at the centre. This was evident when the digital coach provided explanations on relevant topics, as well as when the older person and the digital coach jointly navigated a digital issue in practice. In both cases, the shared information was adapted to the older persons’ needs and level of digital competence. Moreover, new proposals for digital solutions introduced by the digital coach during the support session emerged as shared knowledge adapted to the older persons’ digital competence and digital needs.

When the digital coach provided adapted explanations for digital problems expressed by the older person, opportunities for learning were created.

*“DC: … so if it says that you should use BankID as identification, then this is not BankID, but this is a security token… OP: Is that…? DC: BankID is a bit more like a public ID, but it's like if I'm going into 1177 [digital medical advice and information service in Sweden] and stuff, it's to show my ID… OP: Yes, yes …!”* (Participant 4)

The explanations created opportunities for understanding and for strengthening the older persons’ own digital knowledge, suggesting a form of support through knowledge sharing. Another need for understanding emerged when an older person expressed uncertainty about the internet browsers installed on their computer.

*“OP: There are some … questions of course, it says … browser, which browser do I have, is it Chrome or what is it called? DC: When you go online, … do you use Chrome or what? OP: I'll go with that. DC: Exactly, then … you … have the right one. … Internet explorer, the old version… is not updating”.* (Participant 1)

The question regarding the use of appropriate software created an opportunity to enhance digital competence, as the digital coach shared adapted knowledge about the varying capabilities of different web browsers.

When the support took the form of a joint activity carried out by both the digital coach and the older person, the latter was given the opportunity to develop digital competence through the digital coach’s guided sharing of adapted and contextually relevant knowledge. This is illustrated in the following example, where paper had to be refilled in a printer.

*“DC: … refill paper yes, “place the printer on a hard-flat surface,” we have done that, what are we going to do next… turn the paper tray… the paper conductor… should see …? Well… fill up the A4 letter paper and adjust the paper guides… just… Do you want to try it or should I…? OP: …yes… But will they be pulled out? DC: Yeah, just… OP: Well, that's it. DC: So, you pull them in here too… OP: So, they have been placed… DC: “Close the paper tray,” … the papers have been refilled”.* (Participant 6)

The activities were structured to support the older person in practising and developing digital competence, with the aim of facilitating future independent use based on the adapted knowledge provided by the digital coach.

Sharing adapted knowledge also emerged when the digital coach proposed new digital actions or solutions that had not been addressed earlier in the support session. This is illustrated in the following quote.

*“OP: Well I don't know if I have so much more, if you don't see anything exciting that you would like to trawl with… DC: There are notifications on this one …OP: I've tried to get rid of a lot, but… DC: So there are settings here, so there are notifications and here they are … for example, tips and messages, they are great to send … you have to decide for yourself… OP: Yes, I do, but g-mail is something I don't need to have either…DC: Galaxy, you don't have to either. OP: No, not me anyway”.* (Participant 12)

The quotation suggested enhanced support with the older person’s digital needs in focus. This illustrates how the digital coach actively contributed to knowledge development by introducing new perspectives and solutions, thereby stimulating the participant’s reflection and readiness for action.

## 4 Discussion

### 4.1 Result discussion

The results of this study offer insights into how one-to-one support was provided to older persons in their homes by a municipality-employed digital coach. The findings highlight the importance of supporting digital competence in ways that meet each older person’s individual needs by creating an adapted and inclusive learning environment. The main characteristics of the support that emerged were direct support and memory aid, delivered through hands-on support and the provision of tools to support memory. Building a relationship was essential, along with fostering mutual influence in the support process by creating conditions for equal voice. A foundational characteristic of the support was to place the person’s own digital needs at the centre, achieved through listening and responding and through sharing adapted knowledge.

The premise was that the individual older persons’ varying digital needs, circumstances, and preferences were at focus which was facilitated through home-based one-to-one support. Previous studies have shown that older persons are at different levels of digital competence (3, 17, 27), and the present study confirms the importance of tailoring support to each individual’s digital needs. The initial impression when older persons request support for digital needs may mistakenly be understood as a need for technical assistance only; however, providing appropriate support requires careful attention to their actual needs (18, 39). The present study confirms that the support included many activities beyond technical assistance, such as relationship building, knowledge sharing, listening, and responding.

The digital coach intervention can be viewed as an example of education for lifelong learning (8). Lifelong learning is relevant to all people throughout their lives and serves different purposes. It can exist in various spaces, places and contexts and encompasses formal, non-formal, and informal education (29, 40, 41). The type of lifelong learning described in the present study is non-formal, as the digital coach service is provided by a municipality and delivered in the older person’s home with the intention of supporting digital competence.

Anchored in Fletcher and Mullen’s conceptualisation of coaching as a collaborative and learner-centred process (20), the study identified the provision of digital support for older persons as a coaching-oriented approach. This involves tailoring support to the individual’s level of digital competence, thereby fostering improved digital competence and independence (7, 21). By addressing the need for enhanced digital competence in a population group with below-average levels of such competence, the intervention contributes to bridging the digital divide (2, 3) and countering digital ageism (27).

The characteristics identified in the present study, described through the three overarching themes, illustrate how support was adapted according to older persons’ digital needs. These needs were addressed in relation to the older persons’ individual digital competence, understood through the three levels of the digital divide: access, skills, and meaningful use (2, 3). Receiving direct support and memory aid through hands-on support and tools for memory support enabled the older persons to use their digital devices in ways that met their own needs. Previous studies have identified similar needs for practical support, particularly in relation to older persons’ varying levels of digital competence—such as using digital devices and the internet—as well as the need for tools to help remember pertinent and essential details, especially in the context of age-related memory changes (7, 18, 19). During the support session, both the digital coach and the older person took memory notes, and the latter was encouraged to use a notebook for passwords. Memory support has also been shown to help train cognitive abilities for future digital needs (7, 39), thereby fostering sustained independent use of digital devices beyond the support session. The hands-on support involved both ‘fixing’ digital problems—meaning resolving the issues—and carrying out practical tasks, such as installing an application that the older person wished to use but could not install independently. This hands-on support enabled the older persons to gain access to their own digital devices. Other studies similarly highlight the importance of providing support with digital devices to facilitate access (18, 19). Not having access to functional digital devices relates to the first level of the digital divide, which concerns the basic prerequisite of being able to use digital technology (3).

However, access to functional digital devices alone does not guarantee the ability to use them independently; it also requires digital competence, corresponding to the second level of the digital divide (2, 3). In the present study, hands-on support was supplemented with explanations when deemed appropriate—for example, when the older person received support in understanding the keyboard. Furthermore, the study found that relationship-based characteristics supporting digital competence were essential. These characteristics were realised through relationship-building and by creating conditions that enabled equal voice within the support process. This, in turn, created a permissive learning environment adapted to the individual’s digital needs and current level of digital competence. Other studies have similarly highlighted the importance of creating customised learning environments to promote the development of digital competence among older persons (18, 42). Conversations about the older person’s interests and digital needs contributed to building the relationship and deepening the digital coach’s understanding of the older person’s digital habits. As discussed by Fossum (23), communication between the older person and the digital coach constitutes a crucial component, encompassing both the manner in which the conversation is conducted and the nature of the interaction that develops between them. The importance of relationship-building has also been demonstrated in previous studies as a means of helping older persons overcome barriers to digital competence (7, 43).

Having mutual influence over the support session was manifested through the opportunity to interrupt each other and to change the topic of conversation when needed. This flexibility enabled the older person to develop digital competence during the ongoing support sessions. Flexible learning environments have proven instrumental in overcoming barriers to developing digital competences and improving digital confidence among older persons (21, 44).

In the present study, a central characteristic was identified: to place the older person’s own digital needs at the centre. This characteristic involves communication through conversation, characterised by reciprocal listening and responding. Furthermore, the role of the digital coach encompasses being attentive and sharing knowledge adapted to the person’s needs. Mutual active listening and responsiveness enabled more adopted support for the older person, as illustrated in the results section when the digital coach clarified the meaning of ‘editing a contact’ by using the older person’s own contact list as a reference point. Previous studies have indicated that integrating new information with existing knowledge can enhance comprehension and motivation among older persons, particularly when the content aligns with personally meaningful aspects of daily life (7, 45). The present study also demonstrated that support could be expanded by sharing adapted knowledge relevant to the support session and by focusing on the older persons’ digital needs and their ability and willingness to improve their digital competence.

In this study, the older person’s home emerged as a learning environment conducive to enhancing digital competence, as it is the place where they live their everyday lives and use their digital devices. In this context, digital needs can be adapted to the digital devices available to the older person and to their intended use of these technologies. The non-formal learning environment suggests that both space and place are important for older persons to develop digital competence and digital independence (7, 41). Previous research also highlights the importance of the learning environment in lifelong learning (22). By receiving adapted support with digital needs and devices in the home environment—the space and place where the older person lives and learns in everyday life—older persons are afforded opportunities to develop digital competence in ways that enhance their ability for meaningful use, thereby addressing the third level of the digital divide (3, 29, 41).

### 4.2 Strength and limitations

To answer the aim of the study, a qualitative design with a focused ethnographic approach was used (34). This choice was considered appropriate due to the limited previous knowledge in the research area of digital competence development for older persons (7), a critical concern within public health research. To obtain a holistic understanding and enable in-depth analysis, the researcher (EBK) was present in the room while the support sessions were recorded. This *emic* perspective provided an opportunity to understand the support session in its everyday, natural context. When interpreting the transcribed texts, an *etic* perspective was adopted to contribute to a holistic picture. Gradually, the researcher’s understanding of the digital coach service and the characteristics of the support increased. This pre-understanding was acknowledged and critically reflected upon by the researcher (EBK) to avoid influencing the ongoing support sessions (36, 46). The use of convenience sampling (37) limited the sample to older persons who actively sought support from the municipality’s Digital Coach Service. Although this represents the population of older persons who actually do make use of the service, it may restrict the transferability of the findings to other groups. In line with Braun and Clarke (36, 47) the findings should be understood as situated and shaped by the sampling strategy and the researchers’ involvement in data generation, consistent with a reflexive thematic analytic framework, rather than as methodological biases. To maintain credibility and enable transferability, the sampling process is transparently described in the Method section.

We adopted Braun and Clarke’s rigorous approach as a foundation for ensuring a trustworthy analysis (35). To provide structure and establish trustworthiness—including credibility, dependability, confirmability, and transferability (48)—Braun and Clarke’s reflexive thematic analysis (35, 36) was employed. The analysis process followed the method’s six phases, with a conscious focus on the researcher’s reflexivity to ensure that interpretations were well-grounded in the data.

The study’s credibility was ensured by working systematically and adopting an etic perspective on the text, which involves maintaining an analytical distance to allow for structured interpretation. At the same time, this required reflexive engagement with the researcher’s own role, acknowledging how it shapes the reading of the material. The use of illustrative quotations from the data helped to ground emerging themes in participants’ own voices, enhancing both transparency and depth in the analysis (35, 36, 46), and contributed to the study’s confirmability. Furthermore, in line with Braun and Clarke’s approach to thematic analysis (35, 36), we aimed for dependability by clearly describing the analysis process and for transferability by providing a detailed description of the study context.

A limitation of the study is that all support sessions were conducted by the same digital coach. On the other hand, this offered consistency. Note that, since the participants constitute a group who, for various reasons, chose to seek support for their digital needs, they may differ from the general population of older persons in their willingness to receive digital support. However, the participants did constitute a heterogeneous group in terms of age (68–92 years), cohabitational status, area of residence, and an equal distribution of women and men. While sample size (12 participants) may be regarded as a limitation, this study was guided by its specific aim and prioritised information power over saturation, focusing on analytic depth and relevance rather than representativeness (47). It might also be considered that data collection coincided with the COVID-19 pandemic, which limited opportunities for physical participation and increased reliance on digital solutions in everyday life. However, as digitalisation continues to expand beyond the pandemic, the need for support to enhance digital competence remains (32).

## 5 Conclusion

This study explored how digital support is enacted in real-life interactions when older persons receive one-to-one coaching for digital competence in their homes from a municipally employed digital coach. The results highlight the importance of supporting digital competence in ways that meet each older person’s individual needs and level of competence by creating an adapted and inclusive learning environment. The main characteristics that emerged were direct support and memory aid, support built on relationship and mutual influence, and to place the person’s digital needs at the centre. The digital coach service emerged as a non-formal activity for lifelong learning, promoting opportunities for older persons to develop their digital competence. These insights contribute to a deeper understanding of how digital competence support is enacted and underscore the importance of adapted support to reduce the digital divide and to enable equitable access to digital resources in the digital society.

Future research should examine how these characteristics can inform the design of interventions for supporting digital competence in older persons and whether they can be optimised to enhance effectiveness. For future interventions, the identified themes and their associated sub-themes may serve as an analytical foundation for the development of support aimed at enhancing older persons’ digital competence. This approach is relevant not only for the design and development of municipal services, but also as a resource for informing policy development in this domain. These implications should not be understood as prescriptive solutions, but as analytically grounded directions for future practice and design.

## Data Availability

Data are not publicly available due to ethical consideration and the need to protect participant confidentiality. Data sharing was not approved by the ethical review board. Further queries can be directed to the corresponding author. Requests to access the datasets should be directed to elisabeth.berglund.kristiansson@his.se.
